# Persistence and variation in microstructural design during the evolution of spider silk

**DOI:** 10.1038/srep14820

**Published:** 2015-10-06

**Authors:** R. Madurga, T. A. Blackledge, B. Perea, G. R. Plaza, C. Riekel, M. Burghammer, M. Elices, G. Guinea, J. Pérez-Rigueiro

**Affiliations:** 1 Centro de Tecnología Biomédica. Universidad Politécnica de Madrid. 28223 Pozuelo de Alarcón (Madrid), Spain; 2 Departamento de Ciencia de Materiales. ETSI Caminos, Canales y Puertos. Universidad Politécnica de Madrid, 28040 Madrid, Spain; 3 Department of Biology and Integrated Bioscience Program. The University of Akron, Akron, OH44325-3908, USA; 4 European Synchroton Radiation Facility, B.P. 220, F-38043, Grenoble Cedex, France

## Abstract

The extraordinary mechanical performance of spider dragline silk is explained by its highly ordered microstructure and results from the sequences of its constituent proteins. This optimized microstructural organization simultaneously achieves high tensile strength and strain at breaking by taking advantage of weak molecular interactions. However, elucidating how the original design evolved over the 400 million year history of spider silk, and identifying the basic relationships between microstructural details and performance have proven difficult tasks. Here we show that the analysis of maximum supercontracted single spider silk fibers using X ray diffraction shows a complex picture of silk evolution where some key microstructural features are conserved phylogenetically while others show substantial variation even among closely related species. This new understanding helps elucidate which microstructural features need to be copied in order to produce the next generation of biomimetic silk fibers.

Arthropods produce a great variety of silks from aqueous protein solutions stored in specialized glands. In particular, spiders have up to seven types of silk glands that produce different silks specialized for specific tasks^1^. The toughest fibers of this toolkit are produced from the major ampullate gland (MAS)^2^, and are the constituents of draglines and of the structural elements of most webs. The outstanding toughness of MAS fibers originates from an interplay between amino acid sequence and processing^3^, that is assumed to lead to an optimized microstructural organization. This optimized organization consists of a combination of nanocrystalline and amorphous regions^4^, so that the material simultaneously achieves high tensile strength and breaking strain by taking advantage of weak molecular interactions^5^,^6^. Spider silks are composed of spidroins^7^, a family of large proteins characterized by the presence of a small number of repetitive motifs of amino acid sequences (e.g. A_n_, GA, GGX and GPGX). Each type of silk fiber is differentiated by the identity and abundance of the motifs comprising its core repetitive region, so that differences in composition are assumed to reflect on the microstructure and mechanical properties of the fibers^8^. Intriguingly, and despite strong arguments in favor of accumulation of mutations in spidroin sequences^9^, the basic organization of spidroins was conserved for over 100 million years^10^ in orb-weaving spiders. Elucidating how this design evolved over the 400 million year history of spider silk evolution has proven a difficult task^11^,^12^ largely due to the intrinsic variability of the material, but would provide insight into the key design elements that give spider silks their extraordinary material properties.

X ray diffraction was used to thoroughly characterize the crystalline phase of MAS silk. X ray diffraction patterns were obtained from silks spun by representative species of the four main spider lineages to encapsulate almost the entire history of spider silks^13^ – the Mygalomorphae includes tarantulas and diverged from other spiders prior to the origin of MA silks, the Haplogynae is a basal lineage of spiders with diverse ecologies and relatively simple webs but are among the first spiders to use MA silk for capture webs and draglines to move through their environment, the Entelegynae includes most extant species of spiders and is mostly comprised of two “mega” diverse groups that diverged in how they utilize MA silk. The Orbiculariae use MA silk as the structural backbones of a diverse array of prey capture webs, including the iconic orb web, while species in the RTA clade often lose prey capture webs and instead use MA silk mostly for locomotion^14^. Since the large intrinsic variability of the native material^15^ represents a major drawback for establishing correlations between microstructure and mechanical properties, all analysed samples were initially taken to their ground state^16^ through a maximum supercontraction process^17^,^18^.

Representative XRD patterns of maximum supercontracted fibers are presented in Fig. 1, where the phylogenetic relations between species^19^ are also included. All XRD patterns show proteins organized in antiparallel polypeptide β-sheet structures^20^,^21^ packed into orthorhombic unit cells (Fig. 2a), in accordance with the current view of the structure of β-silk fibroins^12^,^22^. Assuming this geometry, approximately constant values of the inter-planar distances are observed in two perpendicular directions: the inter-chain direction defined by the orientation of the hydrogen bonds between chains forming β-sheets (*a*-direction), and the protein backbone direction (*c*-direction). Both dimensions are similar in all silks and their values are consistent with the β-pleated secondary structure found in β-sheet forming regions which, in turn, make up the crystalline phase^20^. Larger variations are observed in the inter-sheet *b*-direction. The spacings of the main Bragg reflections are shown in Table 1 and the unit cell parameters can be seen in Fig. 2b.

Following Warwicker’s classification of silks^23^, all Entelegynae silks belong to the β(3) group that corresponds to β-sheets of poly-alanine motifs. In contrast, the pattern obtained from *Kukulcania* silk (Haplogynae)^22^ can be assigned to the β(1) group, which is also found in *B. mori* silk. Finally, the pattern obtained from *Aphonopelma* silk (Mygalomorphae) could be assigned to the β(5) group, although its *b* spacing is actually larger than the values indicated originally by Warwicker.

Amino acid sequences of the silk proteins produced by most of the investigated species were taken from previous works^7^,^10^,^24^ and used to establish correlations between primary structure and crystalline microstructure. Spidroin proteins are characterized by the presence of a small number of repetitive motifs of amino acid sequences (e.g. A_n_, GA, GGX and GPGX). Each type of silk fiber is differentiated by the identity and abundance of the motifs comprising its core repetitive region. The main motifs of the spidroin proteins of the analysed fibers are outlined in Table 2.

Mygalomorphae spidroins have a low glycine content and high alanine and serine contents^25^ with a higher proportion of large sidechain amino acids when compared with the silk of other spider species. The main sequence motif in *A. seemani* fibroins is A_n_^25^. However, we hypothesize that the relative scarcity of this motif and the inclusion of bulkier aminoacids in *A. seemani* fibroins explains the increase of the intersheet spacing in the β-polyalanine crystallites^23^. Silk fibers spun by other Mygalomorphae representatives showed a similar tendency to large values of the spacings along the *b*-direction^23^, although the previously reported value of 1.50 nm is slightly lower than that measured from *A. seemani* fibers.

Sequence analysis shows that *K. hibernalis* presents all of the silk motifs that are supposed to form β-sheet nanocrystallites i.e. (GA)_n_, (GS)_n_ and A_n_. However, the XRD analysis clearly shows that *K. hibernalis* diffraction pattern corresponds to the β(1) group^22^ which is characteristic of β-crystallites formed from the (GAGAGS) motif as found in *B. mori* silk^26^. Consequently it is reasonable to think that the crystalline phase in *K. hibernalis* MAS fibers is mainly composed of poly-(GA) or poly-(GS) β-sheet nanocrystallites^27^, as predicted by cDNA data. This hypothesis is also supported by the mechanical properties of the fibers, since *K. hibernalis* silk shows strain-stress curves^19^ which are comparable to those of non-degummed forcibly reeled silkworm (*B. mori*) silk^28^.

All species of the Entelegynae group show XRD patterns compatible with the presence of poly-alanine nanocrystals. The (GS)_n_ motif is consistently absent in all species and the frequency of the (GA)_n_ motif largely reduced. It is worth noticing that the silks of this group, which includes RTA clade and Orbiculariae, share a common unit cell (see Fig. 2b). Since both groups differ in the appearance of the (GPGX) motif in the MaSp2 in the Orbiculariae^10^,^29^, it can be concluded that the presence of the –GPG– motif does not induce any difference in the unit cell of the Entelegynae.

A clear evolutionary trend toward increased formation of β-nanocrystallites from poly-alanine motifs is seen in the Entelegynae silks. This tendency is suggested by the amino acid sequence since Entelegynae silks lack most amino acids with bulky side chains that are common in Mygalomorphae. However, the structure of Entelegynae silks did not result from a simple, gradual loss of these amino acids through homogenization of silk genes. Indeed, β-nanocrystals in *K. hibernalis* (Haplogynae) MA silk are formed primarily from the (GA)_n_ and (GS)_n_ motifs. If these β(1)-nanocrystallites are characteristic of other taxa of basal Araenomorphae (as predicted from cDNAs for a variety of mygalomorphs and haplogynes^25^), then this observation suggests an intermediate stage in the evolution of MA silk nanostructure where β-nanocrystallites were selected for through a variety of amino acid motifs, and only subsequently did strong homogenization of the poly-alanine motif lead to the dominance of β(3)-nanocrystallites during the origin of the Entelegynae. We hypothesize that this could have resulted from either increased functionality (see below) of silk through optimization of β-nanocrystallite size and/or through selection of the metabolic costs of producing these nanocrystals through different amino acid synthesis pathways^12^. The subsequent analysis, however, shows that the basic design principle of imparting structural stability to silk fibers through the formation of a nanocrystalline phase can be implemented in a wide range of microstructural typologies. These typologies, in turn, lead to characteristic variation in the mechanical behavior of silk.

Current models of MA silk consider that the size of the nanocrystals is optimized for improving the mechanical behavior of the material^3^, since larger crystallites would act as effective defects in the material and, consequently, lead to a decrease of toughness^30^,^31^. The sizes of the nanocrystals, as calculated from Scherrer’s equation, are presented in Fig. 2c.

The nanocrystal sizes of the silks from Entelegynae species are very similar–and even similar to that of the Mygalomorphae representative– along all directions and agree with the results obtained in previous studies on other orbicularian MAS fibers^22^,^32^. This similarity also extends to *K. hibernalis* MA silk along the [010] direction, which corresponds to the stacking of the β-sheets.

However, and consistently with the differentiation between β(3) and β(5) poly-alanine, and β(1) poly-glycine/alanine nanocrystals, *K. hibernalis* silk shows larger crystallites along the [100] and, especially, along the [001] directions. The larger size of the nanocrystals along these directions in *K. hibernalis* silk could be related with the presence of the (GA)_n_ and (GS)_n_ motifs in the β-nanocrystals. *Bombyx mori* silk, which also shows a large proportion of (GA)_n_ and (GS)_n_ motifs in its sequence and belongs to the β(1) group, shows comparable large nanocrystals^33^.

The orientation of the crystallites in maximum supercontracted MA silks with respect to the macroscopic axis of the fiber was estimated quantitatively from the full width at half maximum (FWHM) of the Gaussian fitted to the (210) reflection of the azimuthal profile, and the results are presented in Fig. 3a. Except for *A. seemani* silk, orientation generally decreased (FWHM value increased) from basal Haplogynae to the Orbiculariae.

Crystallinity was calculated using the χ parameter^34^ since it is suitable to compare the crystallinity of different silks, although systematically yields values of crystallinity below the percentage of crystallinity obtained with other methods^27^. Figure 3b shows the results obtained for the fibers analyzed. The general trend seems to be that MA silk is more crystalline than ancestral Mygalomorphae silk, but that the degree of crystallinity decreases in more derived taxa –from the highest value, found in *K. hibernalis* (Haplogynae), to lower values in RTA clade and the lowest overall crystallinity found in *A. aurantia* (Orbiculariae).

Finally, the average volume of the individual nanocrystals was calculated from the dimensions w, t and l under the assumption of ellipsoidal geometry as shown in Fig. 3c. The calculation of the volume assuming a parallelepiped shape would follow essentially the same trends, but ellipsoid geometry was preferred since it is the characteristic geometry of the fibers at the nanometer scale observed by atomic force microscopy^35^. Except for *K. hibernalis* silk, no significant differences in the volume of the nanocrystals are observed between different species.

Figure 4a shows the positive relationship between the percentage of supercontraction (%SC)^36^ and crystal orientation. Except for *A. seemani* silk, the orientation of the nanocrystals correlates linearly with the percentage of supercontraction, independently from the type of nanocrystals (R^2^ = 0.66, P = 0.026; R^2^_ic_ = 0.78, P_ic_ = 0.038). The orientation parameter of the maximum supercontracted fibers and the values of breaking strain (Fig. 4b) also correlate positively (R^2^ = 0.66, P = 0.027; R^2^_ic_ = 0.74, P_ic_ = 0.06), as suggested in prior studies^19^. *A. seemani* silk represents an exception to these trends, since it shows no supercontraction and a very low value of the breaking strain, despite yielding a relatively low value for the orientation of the nanocrystals. It is also important to note that *A. seemani* does not possess a MA gland, so that its unique performance is not unexpected.

These correlations are consistent with the current model of supercontraction^37^, since it is assumed that supercontraction of MA silk requires the rotation of the nanocrystals induced by the collapse of the protein-protein hydrogen bonds in the amorphous part of silk^17^,^38^. The microstructural change is enabled by the presence of the (GGX)^39^ motif and increased quantitatively by the presence of (GPGX) motif^40^. Previous works suggest that both motifs might participate in slightly different mechanisms at molecular level^41^ that result in conformational changes of the proteins upon exposure to water. Both effects, however, could not be distinguished from one another with the conventional tensile characterization of supercontracted fibers. In particular, the main differences in the tensile properties of MAS spun by various orbicularian spiders correlates with the content of proline in their MaSp2, which is determined by the number of –GPGX– motifs in the sequence^40^. The data presented above indicate that the largest value of orientation (i. e. lowest value of FWHM) corresponded to *K. hibernalis* MA silk, which does not show significant supercontraction. *K. hibernalis* MA silk exhibits some (GGX) motifs in its sequence, but their rarity compared with the Entelegynae silks^42^ might explain the absence of supercontraction in these fibers. Silks spun by representatives of the RTA-clade and orbicularians –circled in Fig. 4– form consistent groups in Fig. 4a,b, although a more extensive analysis, adding more species will be required to quantitatively test these groups.

The relationship between crystallinity, supercontraction and elastic modulus is explored in Fig. 4c,d. Crystallinity does not correlate with the percentage of supercontraction (Fig. 4c). In contrast, increased crystallinity does predict higher elastic modulus (Fig. 4d; R^2^ = 0.99, P = 0.0002; R^2^_ic_ = 0.89, P_ic_ = 0.007) which, notably, is independent of phylogeny and of the type of nanocrystals. The strength of this relationship is congruent with current models that explain the initial mechanical behavior of spider silk arising from the combination of a hydrogen bond lattice and β-nanocrystallites^6^ in a way that is largely independent from other microstructural features.

The volume of individual nanocrystals did not correlate with supercontraction (Fig. 4e) or toughness (Fig. 4f). In both cases, the β(5) poly-alanine nanocrystals of *A. seemani* and the β(1) poly-glycine-alanine nanocrystals of *K. hibernalis* are clear outliers. No clear trend, however, could be established from the comparison of silks spun by the RTA-clade and Orbicularian representatives.

These results establish links between the structure of silk and its functional properties. β-nanocrystals act as the element that provides silk with its structural integrity, presumably since the origin of MA silk. Initially the crystalline phase consists primarily of polyalanine nanocrystals, which likely arose from upon a variety of amino acid motifs including the –GA– motif found in Haplogynae, and selection subsequently led to the small and uniform size of the β-nanocrystals found in Entelegynae MAS. A comparable conservation in the basic organization of the amino acid sequence of the spidroins is observed when different Entelegynae species are analyzed^10^, despite their divergence for over 230 million years. These findings suggest the existence of strong evolutionary pressures maintaining some basic traits of spider silk, such as the dimensions of the nanocrystals, close to their optimal values^30^. The extreme conservation of sequence and microstructure in spider silk is even more striking since it contradicts initial hypotheses that considered a significant accumulation of mutations in these proteins as very likely^9^.

In addition to the stability derived from the highly controlled dimensions of the β-nanocrystals, high toughness (where is W_f_ ≥ 50 MJ/m^3^, and often exceeds Kevlar) seems to stem from two additional microstructural features of the nanocrystals: decrease in the orientation of the nanocrystals in the maximum supercontracted state and, possibly, reduction in the crystallinity. The lower orientation and a tendency towards reduced crystallinity observed in Orbicularians compared to other spiders may be explained in part by the expression of a second type of spidroin (MaSp2) in their silk with its GPGX amino acid motif that is predicted to disrupt crystallinity^43^. Indeed, in our study *Argiope aurantia* MA silk, which exhibited the maximum value of work to fracture of all species^19^, also showed the lowest values of the orientation of the nanocrystals and of crystallinity. The previous analysis shows that decreased orientation and, possibly, decreased crystallinity correlate with higher values of supercontraction, strain at breaking and, correspondingly, toughness. Consequently, it can be hypothetized that the optimization of these parameters during evolution might be the key selective pressure that modeled the microstructural organization of MA silk. These results further suggest that creating a microstructure of small and uniform nanocrystals in an amorphous matrix that allows a significant conformational freedom to the constituent proteins during stretching might be the base of new families of high performance biomimetic fibers.

## Methods

### Taxon selection and collection of silk

Seven different spider species spanning four major events in the evolution of dragline silk are included in the study:

The tarantula *Aphonopelma seemani* represents the Mygalomorphae, the likely sister taxon to the Araneomorphae, which includes the earliest known MA producing spiders. Tarantulas and their allies diverged from Araneomorphae at least 390 MY ago^9^. These spiders do not produce MA silk, but the relatively undifferentiated fibers offer the best comparison to MA silk spun by Araneomorphae. cDNA libraries show that their silk proteins are relatively unspecialized and mostly lack the functional motifs found in other spiders’ silks. The spider was purchased from the pet trade (Tarantulaspiders.com).

*Kukulcania hibernalis*, collected in Florida (USA), represents the Haplogynae, a basal group that diverged ~375 MY ago from others araneomorphs, soon after the origin of the MA silk gland. cDNA libraries show that *K. hibernalis* MA proteins are relatively unspecialized compared to the other evolutionarily derived lineages in our investigation^10^.

**Figure 1:**
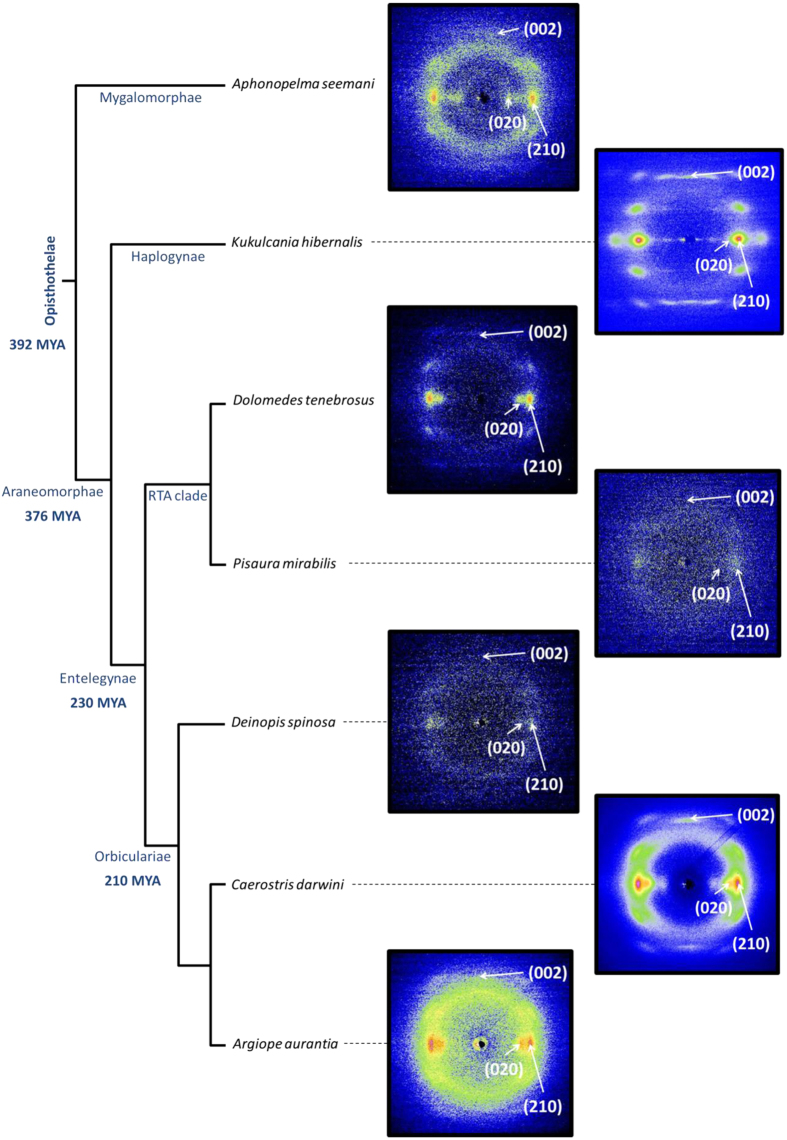
Representative XRD spectra of maximum supercontracted MAS fibers of the analysed species represented in a phylogenetic tree^19^. The most significant Bragg’s peaks are shown in each XRD pattern.

All other species belong to the Entelegynae, which is broadly characterized by a significant increase in the repetitiveness of the amino acid sequences in their MA proteins. The RTA clade (wolf spiders, jumping spiders and their allies) diverged from the Orbiculariae, approximately 210 MY ago, prior to the origin of orb webs. This divergence was thought to have occurred prior to the origin of orb webs, but recent phylogenomic analyses suggest that RTA clade spiders fall within traditional Orbiculariae, so that either the orb web evolved multiple times or the RTA clade evolved from an orb-weaving ancestor^14^,^44^.Regardless, the two groups represent very different ecological pressures on how MA silk is used –primarily as lifelines and a structural element of sheet webs in the RTA clade or as key structural elements of orb webs in the Orbiculariae. Both of these functions require MA silk to strongly dissipate mechanical energy it absorbs, leading to fibers with several similar aspects in their mechanical behavior^19^. However, energy dissipation in orb webs is facilitated by strongly non-linear stress-strain characteristics in MA silk^45^. *Dolomedes tenebrosus* and *Pisaura mirabilis* represent the RTA clade and were collected in Bath (OH). *Deinopis spinosa* was collected in Gainesville (Fl) and utilizes cribellate silk in its web – a type of dry adhesive that was subsequently replaced by viscid glues in all other orb weavers–. *Caerostris darwini* was collected from Andasibe-Mantadia National Park (Madagascar) and *Argiope aurantia* was collected in Bath (OH). The phylogenetic relationship shown in Fig. 1 is taken from^19^ – recent phylogenomic studies differ only in placing *D. spinosa* ancestral to RTA clade. Silk was collected from most spiders using forced silking^2^. Spiders were restrained on petri dishes, but conscious, and silk was drawn from the spinnerets at a speed of 2 cm/s. The spinnerets were visualized under a stereomicroscope to ensure that only threads originating from the major ampullate gland were collected. Both *Aphonopelma* and *Kukulcania* rarely produce threads forcibly so they were instead walked across fluted sheets of cardboard so that their draglines could be collected. In most cases, the silk used for the XRD analysis was retrieved from a single specimen – in some, but not all cases, these were the same spiders used to retrieve silk for the mechanical characterization as published previously^19^. Silk was mounted across 15 mm long gaps on cardboard holders and secured with cyanoacrylate.

### Maximum supercontraction of silk

Fibers were cut from the cardboard holders and mounted on aluminum foil frames. The gauge lengths of the samples were approximately 10 mm, determined with a profile projector (Nikon V-12B; resolution ± 10 μm). The samples were mounted in a tensile testing machine (Instron 4411) and the length at which the fiber was taut, but not subjected to load was determined – this length is called zero load length (L_0_). Then the fiber was left slack and, under this condition, immersed in water^16^. After removal from water the fiber was allowed to dry overnight. It was checked that the fiber remained slack after drying and subsequently the new zero load length was determined. This length is called length of maximum supercontraction (L_MS_), and the percentage of supercontraction is defined from L_0_ and L_MS_ as: %SC = (L_0_ – L_MS_)/L_0_ × 100. Usage of maximum supercontracted fibers for both microstructural analysis and mechanical characterization is critical to reduce the uncertainty of the results, since the maximum supercontracted state is a property characteristic of each species. In this regard, the same maximum supercontracted state is reached independently from the specimen and from the previous loading history of the sample.

### XRD data collection and analysis

Fibers were transferred and held taut in an adapted aluminum frame, so that the fiber axis was oriented perpendicular to the X-ray beam. XRD images were obtained using synchrotron radiation at the ESRF-ID13 in Grenoble (France) using a monochromatic beam from crossed mirrors of size ≈1 × 1 μm^2^ and a wavelength of 0.1 nm, as explained in detail elsewhere^27^. At least two fibers were analyzed for each species. A scan of 256 images was performed on each sample including patterns inside and outside the fibers. Patterns recorded outside the fiber were averaged and subtracted from the average of patterns recorded inside the fiber. XRD data were used to study the unit cell of the fibers, their crystallinity, the sizes of the nanocrystals and their orientation with respect to the macroscopic axis of the fiber. All these analyses were performed with the FIT2D program^22^. Despite careful sample preparation some sources of variation among fibers of the same species remain as shown by the error bars found in the analysis of the different microstructural parameters. The main sources of variation are probably related with the difficulty of manipulating the material and getting accurate measurements with tolerances below 1 μm. As indicated above, the systematic usage of maximum supercontracted fibers and synchrotron radiation keeps errors low enough to drawing reliable conclusions from the data.

### Unit cell and particle sizes

The main diffraction peaks in all cases were compatible with the reflections (020), (210) and (002) of an orthorhombic unit cell^23^. The unit cell was calculated from the distances between these peaks. XRD patterns were azimuthally integrated resulting in a 1D profile that represented intensity versus reciprocal lattice spacings (Q). The 1D profile was fitted with Gaussian functions: one for each Bragg reflection and an additional one for the short-range-order peak^27^, as well as a constant for the residual background scattering of the sample. The size of the particles (L) was determined by Scherrer’s equation, L = (0.9λ)/(Bcosθ), where B is the radial width (fwhm) of the Gaussian fitted to the diffraction spot^27^.

### Orientation and crystallinity

The orientation of the nanocrystals with respect to the fiber axis was determined from the azimuthal broadening of the (210) equatorial reflection^34^. The diffraction pattern was radially integrated on a thin azimuthal section containing the (210) reflection. The 1D profile obtained after integration was fitted using Gaussian functions and a constant. The orientation of the nanocrystals has been quantified by the full width at half maximum (FWHM) of the Gaussian used to fit the (210) reflection.

The crystallinity of silk fibers has been quantified from different indexes in previous studies^23^,^27^,^34^. Due to the heterogeneity of the samples analyzed in this study a method that considers the whole pattern was chosen. In this method, the intensity of the whole diffraction pattern, *I*_*T*_*(Q),* is determined as a function of the magnitude of the scattering vector *Q* *=* *4πsin(θ)/λ*. The azimuthally averaged intensity of the amorphous halo, *I*_*A*_*(Q)*, can be calculated by azimuthal integration in selected regions, where it is considered that the intensity arising from Bragg peaks is negligible. The intensity of the Bragg peaks is then obtained as: i_c_(Q) = *I*_*T*_*(Q) – I*_*A*_*(Q)*. The ratio between the integrated intensity of Bragg peaks and the integrated total intensity defines the χ parameter^27^,^34^ as:


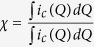


### Statistical analyses

Mechanical properties were correlated with silk structure using general regression models in Statistica 10 (StatSoft, Inc. Tulsa, OK, USA). Each species’ average performance and structural properties were used as the primary data. A polynomial model was used in instances where it provided a significantly better fit than a linear model (e.g. between initial modulus and % crystallinity).

## Additional Information

**How to cite this article**: Madurga, R. *et al.* Persistence and variation in microstructural design during the evolution of spider silk. *Sci. Rep.* **5**, 14820; doi: 10.1038/srep14820 (2015).

## Supplementary Material

Supplementary Information

## Figures and Tables

**Figure 2:**
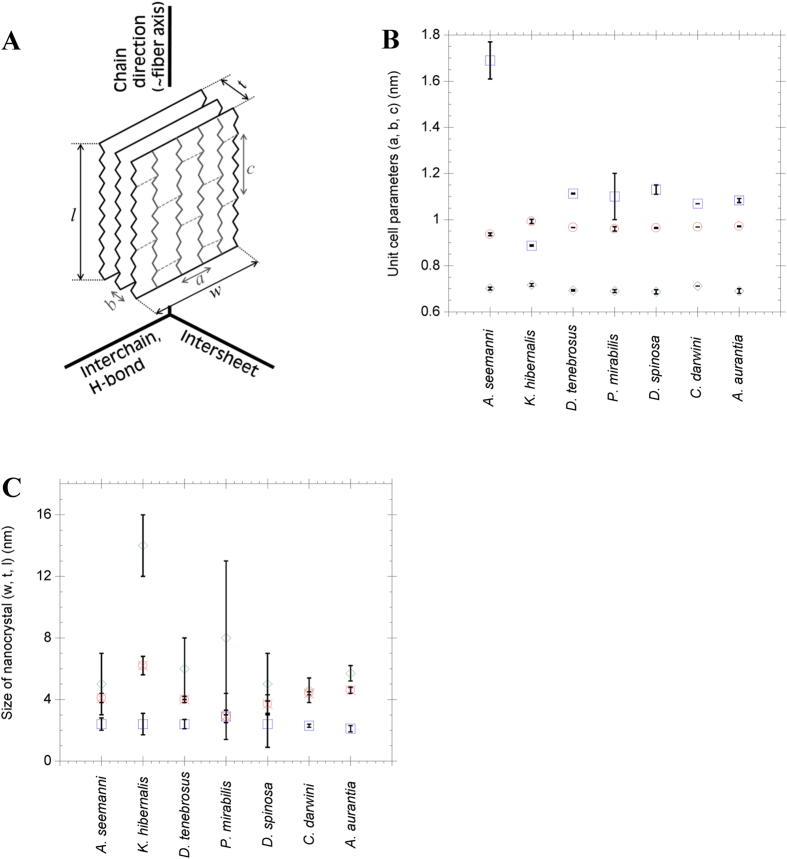
(**A**) Schematic representation of a β-sheet nanocrystal showing the directions of the unit cell and the labelling used to describe the unit cell and nanocrystal dimensions. (**B**) Representation of the unit cell dimensions for the different spider species. Circles are for *a*-dimension (interchain direction), squares correspond to *b*-dimension (intersheet direction) and diamonds are for *c*-dimension (protein backbone direction). Error bars represent standard error. (**C**) Particle size of the nanocrystallites in the [100] (crossed circles, dimension *w*), [010] (squares, dimension *t*) and [001] (diamonds, dimension *l*) directions. Error bars represent standard error.

**Figure 3:**
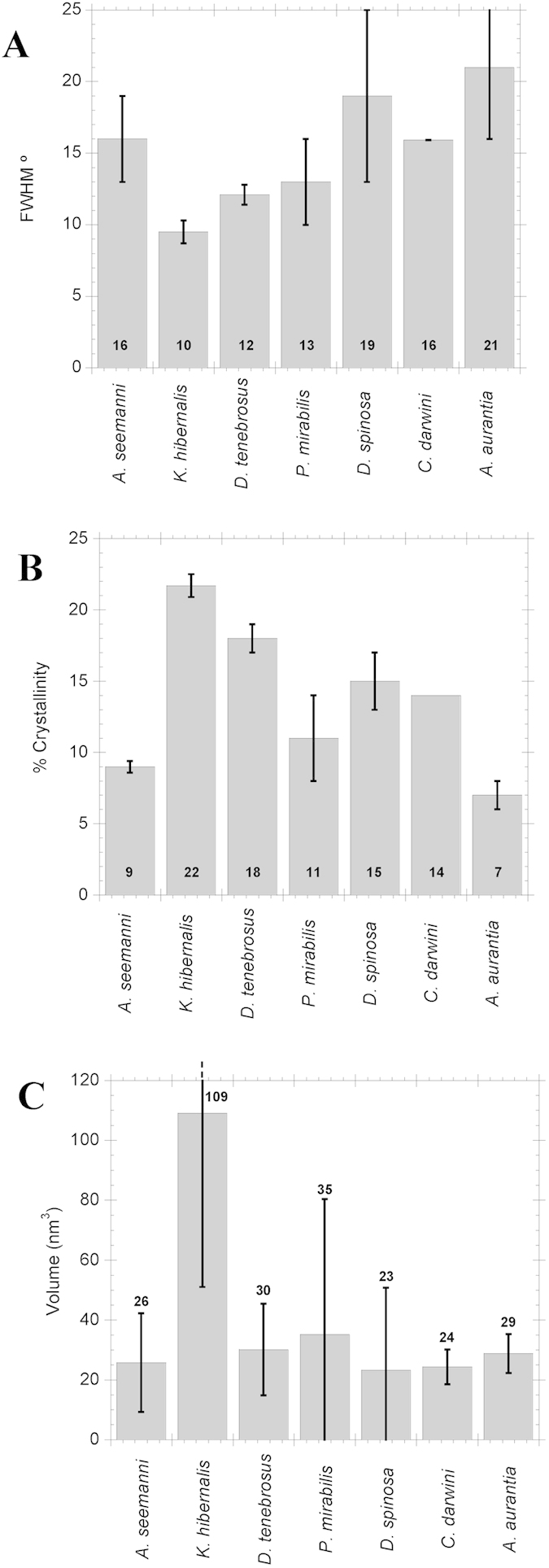
(**A**) Mean orientation of the β-sheet nanocrystals with respect to fiber axis, quantified from the FWHM of the Gaussian functions obtained from the fitting of azimuthal intensity at (210) reflections. Higher values of FWHM mean less orientation of the nanocrystals with respect to the fiber axis. (**B**) Crystallinity of the fibers calculated as χ. (**C**) Volume of the nanocrystals for the different species obtained from the apparent particle size (see Fig. 3) assuming ellipsoid geometry. The number in each bar indicates the mean value of the parameter and error bars represent standard error. All data, except for *C. darwini*, where n = 1, are the result of averaging the corresponding parameter from at least two different fibers of the same species.

**Figure 4:**
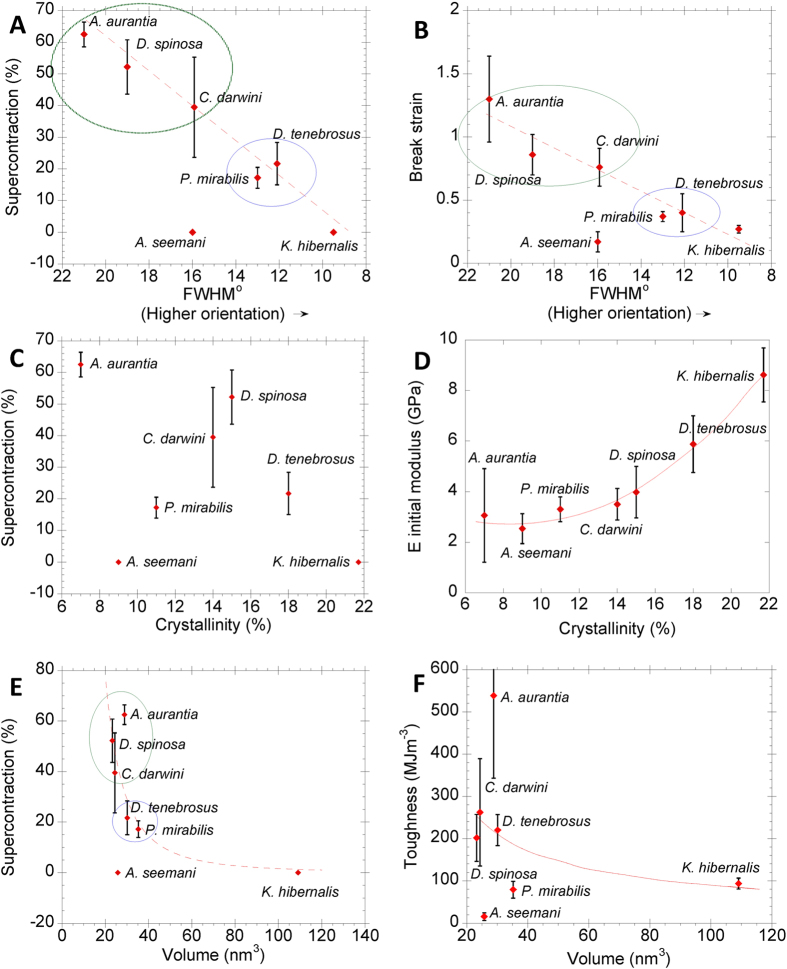
(**A,B**) Orientation, measured as azimuthal FWHM of the (210) reflection, versus supercontraction and strain at breakage, respectively. Supercontraction (**C**) and elastic modulus (**D**) as a function of crystallinity. Supercontraction (**E**) and toughness (**F**) as a function of the volume of the individual nanocrystal. Error bars represent the standard error as calculated from the mechanical data found in^19^. Error bars along the X axes are not shown to improve the clarity of the plots but can be readily inferred from Fig. 3.

**Table 1:** Spacings of main Bragg reflections.

Species	Distances (Å)				Proposed group^1^
	(100)	(020)	(210)	(002)	
*A. seemani*	—	8.7 ± 0.2	4.51 ± 0.06	3.48 ± 0.04	~β(5)
*K. hibernalis*	9.5 ± 0.7	4.6 ± 0.1	4.4 ± 0.1	3.58 ± 0.03	β(1)
*D. tenebrosus*	9.5 ± 0.7	5.5 ± 0.1	4.43 ± 0.03	3.47 ± 0.01	β(3)
*P. mirabilis*	9.1 ± 0.7	5.3 ± 0.5	4.4 ± 0.1	3.53^2^	β(3)
*D. spinosa*	10 ± 1	5.6 ± 0.3	4.5 ± 0.1	3.47 ± 0.05	β(3)
*C. darwini*	9.0 ± 0.4	5.3 ± 0.2	4.4 ± 0.1	3.525 ± 0.009	β(3)
*A. aurantia*	9.3 ± 0.1	5.4 ± 0.2	4.4 ± 0.1	3.44 ± 0.05	β(3)

^1^Groups are labelled following Warwicker’s classification. The pattern of *A. seemani* was assigned to β(5) group despite the value of the b-dimension is actually larger than that originally proposed by Warwicker.

^2^Due the noisy images of this sample the (002) reflection has been analyzed only in one pattern.

**Table 2:** Sequence motifs present in the analyzed species and percentage of small aminoacids.

Motif	*A. seemani*^25^	*K. hibernalis*^42^,^46^	*D. tenebrosus*^10^	*D. spinosa*^46^,^47^	*A. aurantia*^10^
A_n_	✓	✓	✓	✓	✓
(GS)_n_		✓			
(GA)_n_		✓	✓	✓	
(GGX)_n_		✓	✓	✓	✓
GPG(X)_n_				✓	✓
(G+A+S)%	34	78	72	69	70

Analyses were performed from sequences available at the National Center for Biotechnology Information (http://www.ncbi.nlm.nih.gov/protein; Release May 1^st^ 2014). No sequence for the fibroins of *P. mirabilis* or *C. darwini* were available at the time of writing this work.
