# Supplementary material for: Persistence and variation in microstructural design during the evolution of spider silk
Source: Sci Rep. 2015 Oct 6;5:14820. doi: 10.1038/srep14820 (PMC4594040; doi:10.1038/srep14820)
Supplement: Supplementary Information [file srep14820-s1.pdf]

Persistence and variation in microstructural design during the evolution of spider silk

R. Madurga[1,2], T.A. Blackledge[3], B. Perea[1,2], G.R. Plaza[1,2], C. Riekel[4], M. Burghammer[4], M. Elices[1,2], G. Guinea[1,2], J. Pérez-Rigueiro[1,2*]

[1] Centro de Tecnología Biomédica. Universidad Politécnica de Madrid. 28223 Pozuelo de Alarcón (Madrid). Spain

[2] Departamento de Ciencia de Materiales. ETSI Caminos, Canales y Puertos. Universidad Politécnica de Madrid, 28040 Madrid. Spain

[3] Department of Biology and Integrated Bioscience Program. The University of Akron, Akron, OH44325-3908.USA

[4] European Synchroton Radiation Facility, B.P. 220, F-38043, Grenoble Cedex. France

SUPPLEMENTARY INFORMATION

FIGURE METHODS

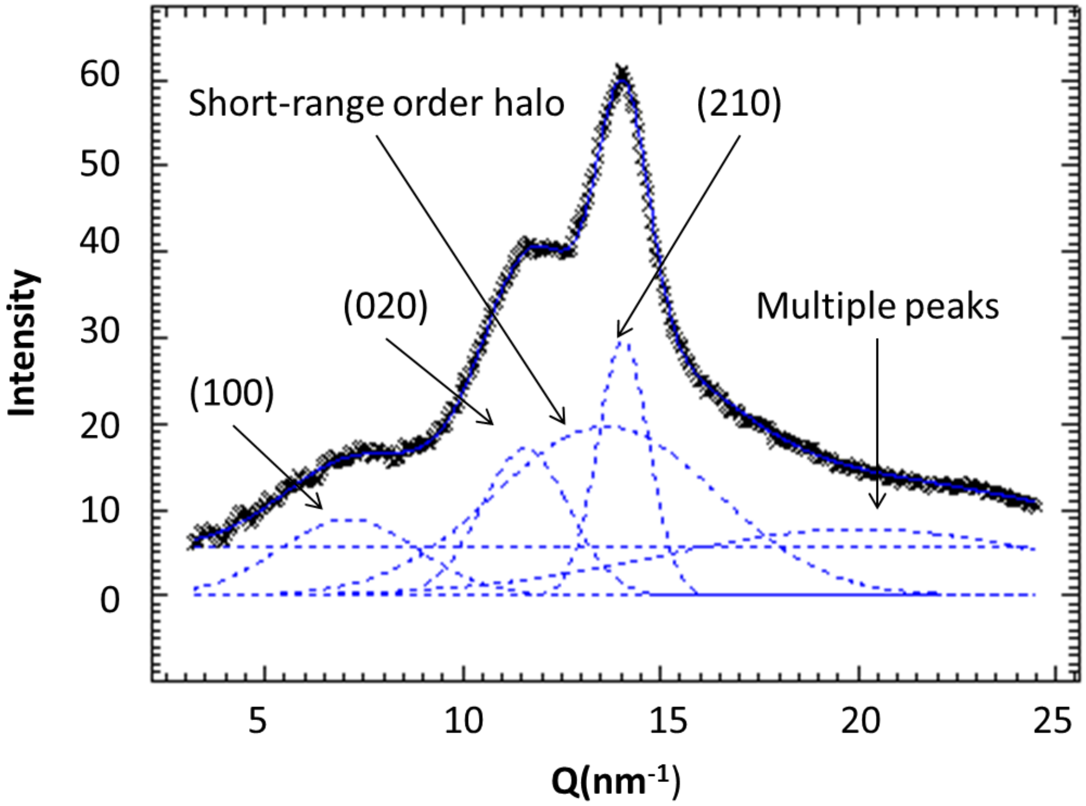

Figure Methods 1: Representative azimuthal integration of an XRD diffraction pattern, and deconvolution into different contributions. The experimental profile is fitted using Gaussian functions for the Bragg reflections and short-range-order scattering, and a constant for the residual background scattering of the sample. Experimental data correspond to a maximum supercontracted fiber spun by a *Caerostris darwini* spider.

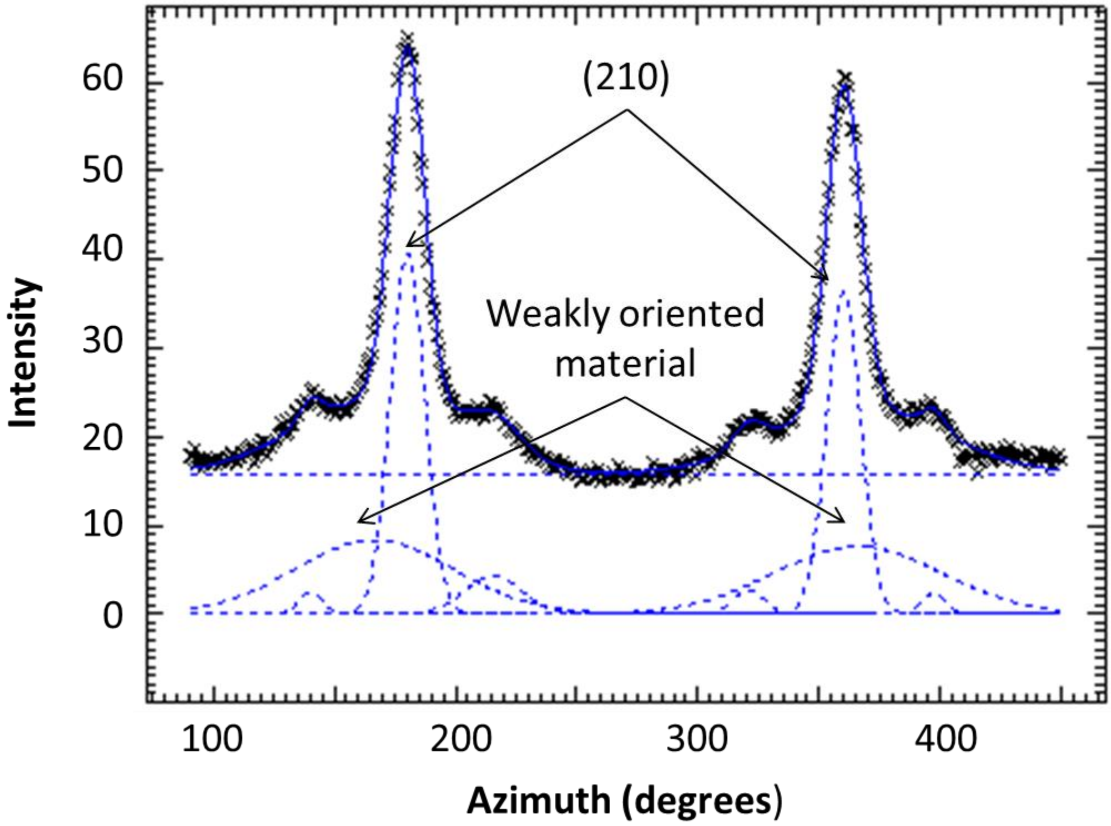

Figure Methods 2: Representative azimuthal fit of the radially integrated (210) reflection from an XRD pattern of a maximum supercontracted MAS fiber spun by a *Caerostris darwini* spider assuming Gaussian functions for the Bragg reflections and a constant background.

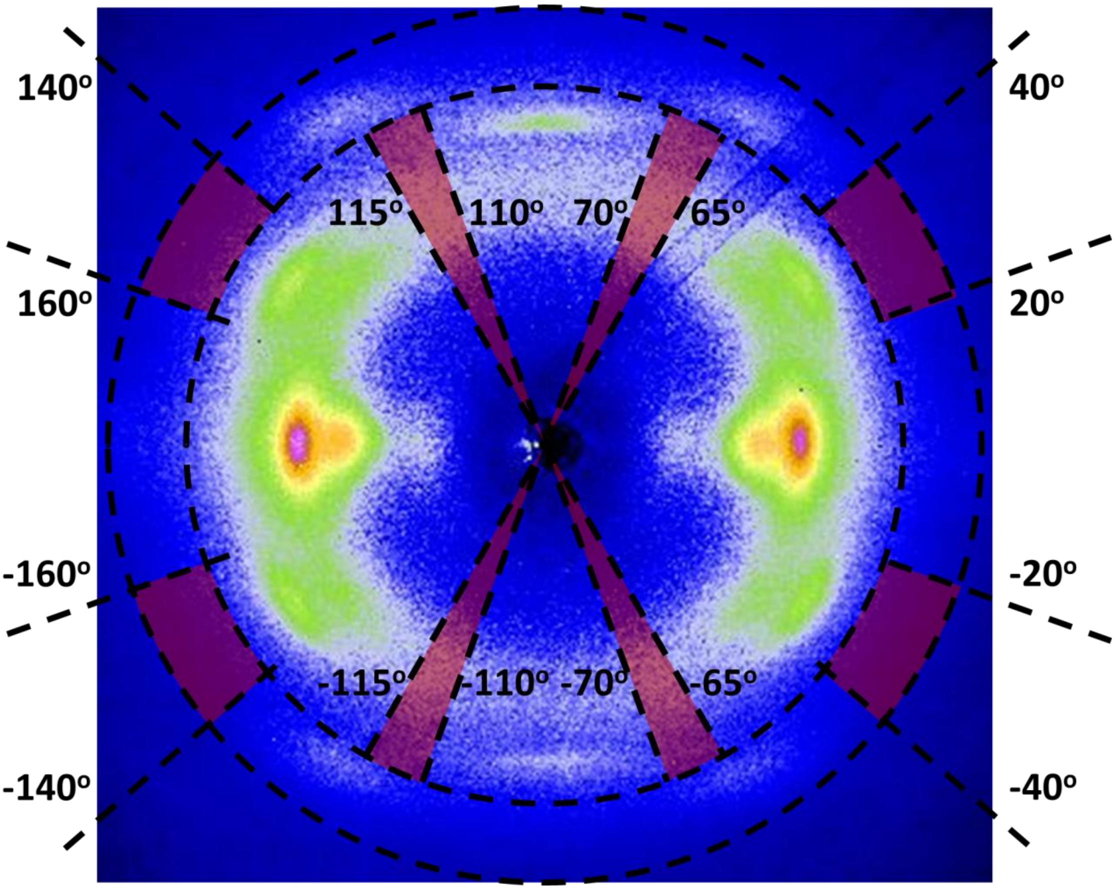

Figure Methods 3: Scheme of the areas selected to calculate the average intensity produced by the amorphous fraction of the fiber by azimuthal integration. XRD pattern corresponds to a *Caerostris darwini* MAS fiber. Angles were adjusted, if necessary, for other patterns in order to select areas where the intensities of the Bragg peaks can be considered negligible.
